# Comprehensive landscape and future perspectives of non-coding RNAs in esophageal squamous cell carcinoma, a bibliometric analysis from 2008 to 2023

**DOI:** 10.3389/pore.2024.1611595

**Published:** 2024-02-21

**Authors:** Jiaxin Wu, Yuanying Wang, Yi Cheng, Li Cheng, Lushun Zhang

**Affiliations:** ^1^ Graduate School, Chengdu Medical College, Chengdu, China; ^2^ Department of Radiology, People’s Hospital of Lushan County, Ya’an, China; ^3^ Department of Pathology and Pathophysiology, Chengdu Medical College, Chengdu, China

**Keywords:** novel biomarker, non-coding RNAs, digestive system cancer, bibliometric analysis, VOSviewer

## Abstract

**Objectives:** Summarize the progress and hot topic evolution of non-coding RNAs (ncRNAs) research in esophageal squamous cell carcinoma (ESCC) in recent years and predict future research directions.

**Methods:** Relevant articles from the Web of Science until 31 October 2023 were obtained. Bibliometric analysis of included articles was performed using software (VOSviewer, CiteSpace, and Bibliometrix). The volume and citation of publications, as well as the country, institution, author, journal, keywords of the articles were used as variables to analyze the research trends and hot spot evolution.

**Results:** 1,118 literature from 2008 to 2023 were retrieved from database, with 25 countries/regions, 793 institutions, 5,426 authors, 261 journals involved. Global cooperation was centered on China, Japan, and the United States. Zhengzhou University, an institution from China, had the highest publication. The most prolific author was Guo Wei, and the most prolific journal was Oncology Letters. Analysis of keywords revealed that the research in this field revolved around the role of ncRNAs in the occurrence, development, diagnosis, treatment, and prognosis of ESCC, mainly including micro RNAs, long non-coding RNAs, and then circular RNAs.

**Conclusion:** Overall, research on ncRNAs in ESCC remains strong. Previous research has mainly focused on the basic research, with a focus on the mechanism of ncRNAs in the occurrence, development, diagnosis, treatment, and prognosis of ESCC. Combining current research with emerging disciplines to further explore its mechanisms of action or shifting the focus of research from preclinical research to clinical research based on diagnosis, treatment, and prognosis, will be the main breakthrough in this field in the future.

## Introduction

Esophageal squamous cell carcinoma (ESCC) is a highly aggressive malignancy with the seventh incidence rate and a leading cause of cancer-related deaths around the world [1]. Despite significant advancements in surgical techniques, chemotherapy, and radiation therapy, as well as the emergence of new targeted therapy and immunotherapy, the five-year survival rate of ESCC patients remains low at only 10%–25% [2]. Thus, the identification of novel biomarkers and therapeutic targets is essential. Non-coding RNAs (ncRNAs) constitute 98%–99% of the transcriptome, and do not encode proteins. Instead, they play crucial roles in various biological functions [3]. In recent years, ncRNAs have emerged as promising diagnostic and prognostic biomarkers, and as potential therapeutic targets in ESCC, and the most widely studied ncRNAs are microRNAs (miRNAs), long non-coding RNAs (lncRNAs) and circular RNAs (circRNAs). These ncRNAs are abnormally expressed in ESCC and participate in various biological functions such as gene expression, cell cycle, and epithelial-mesenchymal transition (EMT) through multiple mechanisms, promoting the occurrence and development of ESCC [4–6].

Bibliometrics is a method of quantitative analysis and evaluation based on the use of literature database records. It is employed to analyze the production efficiency and characteristics of publications in a particular field, assess the quality and impact of academic outcomes, and forecast trends and directions [7, 8]. So far, bibliometric has been widely used in the fields of biosciences and clinical medicine and has provided a new horizon for research in related fields [9–11].

Currently, there has been a large amount of previous research on ncRNAs associated with ESCC worldwide, and numerous research achievements have been obtained. However, the research progress and hot spot evolution in the field are still unclear. Therefore, a comprehensive bibliometric visualization analysis of the research on ncRNAs in ESCC is warranted. This study analyzed previous studies on ncRNAs associated with ESCC using bibliometric approach and visualized the results in an effort to gain insight into the past and current research status, identify potential directions for the future research in this area.

## Methods

### Literature acquisition and data collection strategy

**FIGURE 1:**
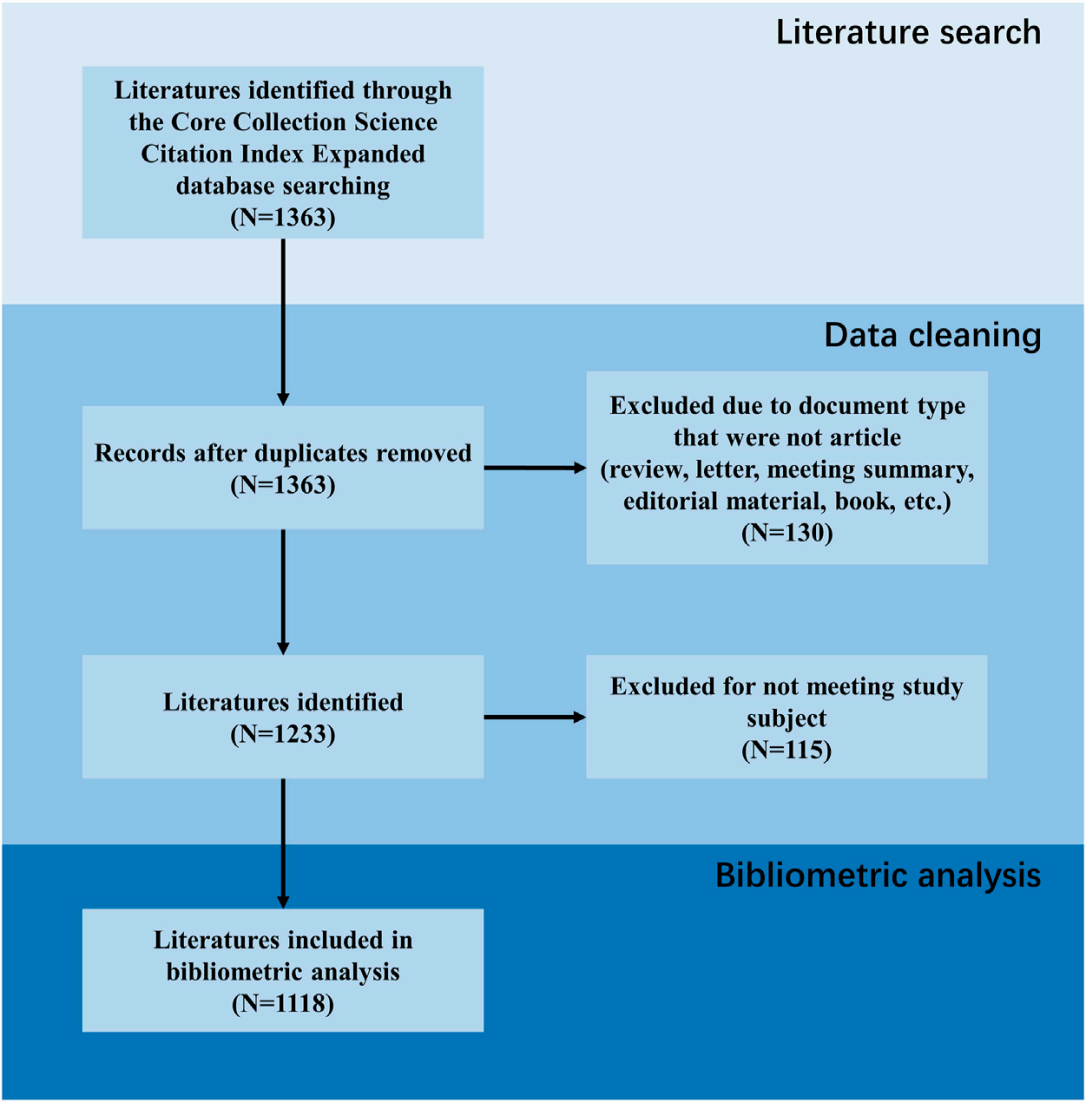
Flow diagram of literature inclusion.

To guarantee comprehensive and accurate data retrieval, literature was searched in the Web of Science Core Collection Science Citation Index Expanded (SCIE) database until 31 October 2023 and exported as “plain text” format. Two reviewers conducted a systematic literature search independently. A senior investigator made final decisions concerning any discrepancies between the two independent reviewers. As shown in Figure 1, after obtaining literature from database, data cleaning was carried out. Duplicate documents were first excluded, followed by document type that were not articles (including review, letter, meeting summary, editorial material, book, etc.). Finally, the content of the literature was reviewed and those that did not meet the research theme were also excluded. The search formula was as follows:

**#1**: TS = (“esophageal squamous cell carcinoma” OR “oesophageal squamous cell carcinoma”).

**#2**: TS = (“non-coding RNA” OR “noncoding RNA” OR “ncRNA” OR “microRNA” OR “miRNA” OR “small RNA” OR “snoRNA” OR “snRNA” OR “long non-coding RNA” OR “lncRNA” OR “circular RNA” OR “circRNA” OR “piwi-interacting RNA” OR “piRNA”).

**#3**: **#1** AND **#2**.

### Bibliometric analysis and visualization

We mainly used VOSviewer (ver. 1.6.19), CiteSpace (ver. 6.1.R 2), and R package Bibliometrix (ver. 4.1.2) for the analysis and visualization of the retrieved data [12]. Annual volume of publications as well as keywords with the strongest citation burst were obtained using CiteSpace. Publication information of the source journals and high-citation articles were obtained using VOSviewer. The co-authorship analysis of country/region, organization, and author were analyzed and visualized using VOSviewer. A world map enabling visualization of the volume of publications and cooperation between countries was drawn using R package Bibliometrix. After manually standardized keywords with different spellings but same meanings, the 100 keywords with the highest frequency of occurrence were obtained using VOSviewer and visualized according to the strength of association and average year of occurrence.

## Results

### The annual trend in publications

We obtained literature from the SCIE database according to the developed search strategy and removed records that did not meet the inclusion criteria. The above process is presented in Figure 1, identifying a total of 1,118 articles included in this study.

**FIGURE 2:**
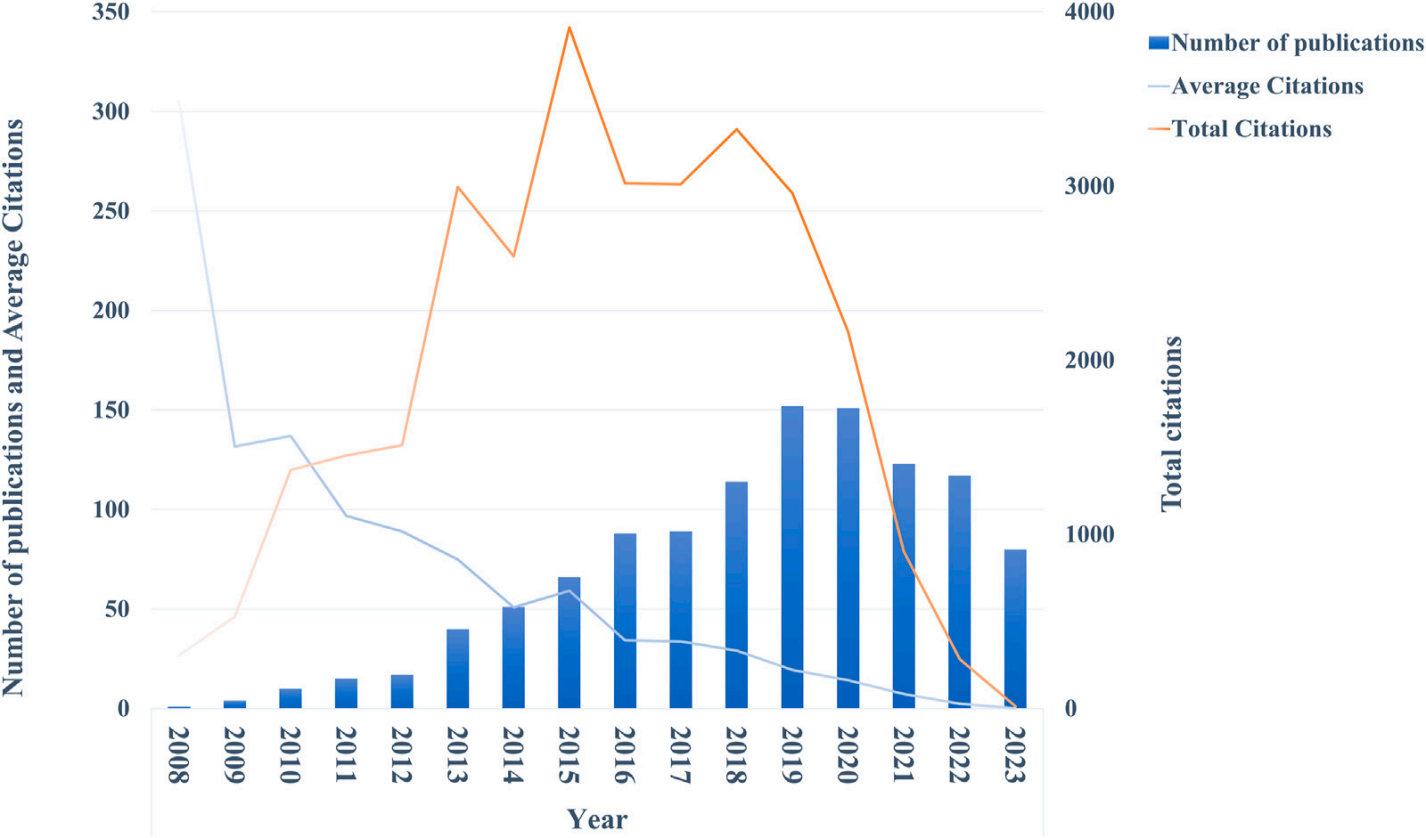
Annual publications trend chart.

As shown in Figure 2, over the past 15 years, there had been an annual increase in publications on ncRNAs associated with ESCC. Since the first publication in 2008, the annual publications had begun to climb. In 2014, the number breached 50, and in 2018 it surpassed 100. The peak was reached in 2019 with 152 publications. However, the volume of publications declined in the following 3 years compared with 2019. In addition, the increasing trend of total citations was not as robust as the volume of publications, and it showed a decreasing trend after peaking in 2015. Notably, the mean citation of papers in the field was high at first but decreased year after year, apart from a transient rise in 2010 and 2015.

### The co-authorship analysis of countries and organizations

**FIGURE 3:**
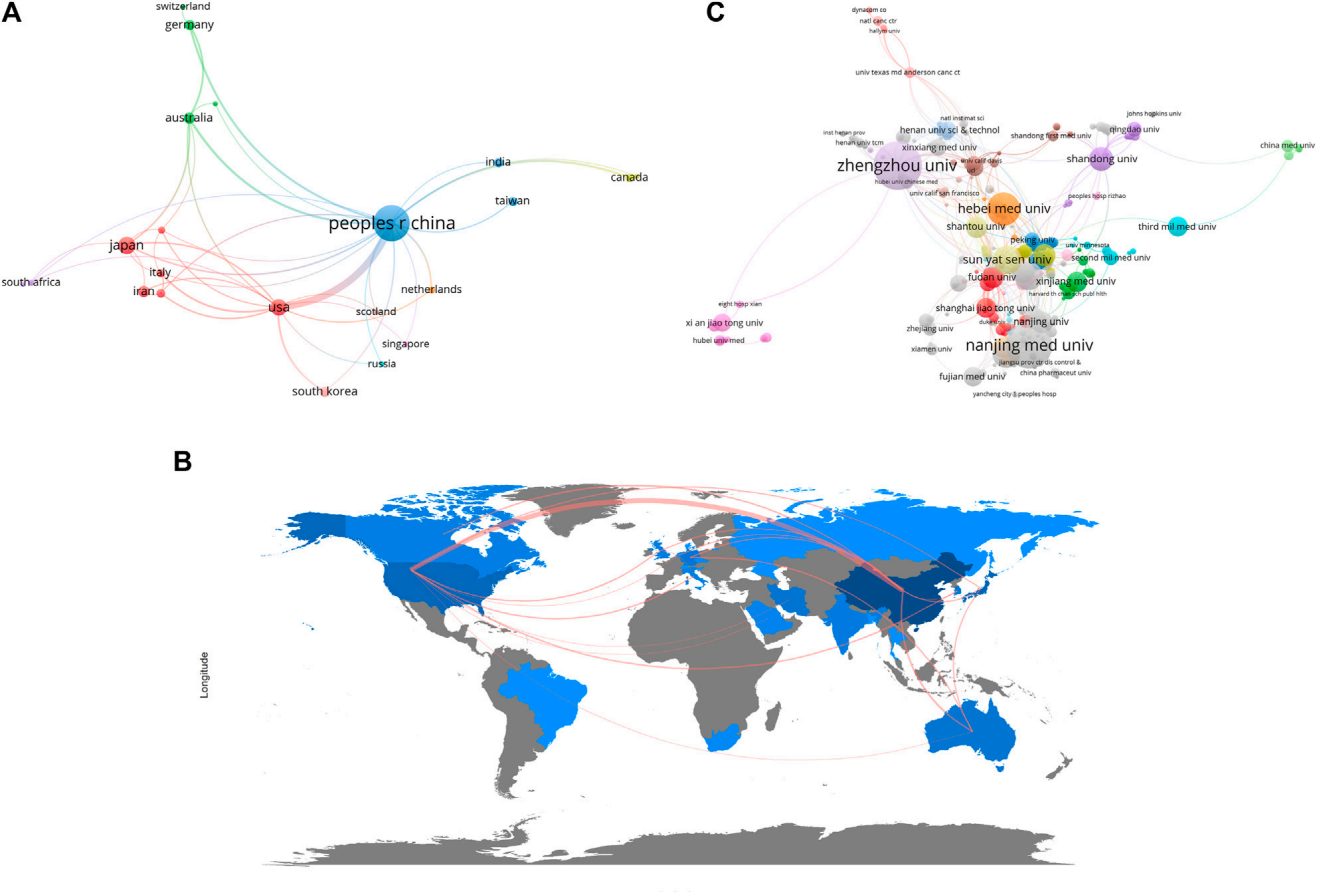
The co-authorship analysis of ncRNAs associated with ESCC. **(A)** Network plot of co-authorship analysis of countries/regions. **(B)** World map of co-authorship analysis of countries/regions. **(C)** Network plot of co-authorship analysis of organizations. The size of the circles or the depth of map color blocks represents the number of publications, and the thickness of the lines between the circles or the map color blocks represents the degree of collaboration.

There were 25 countries/regions that have conducted relevant research, Supplementary Table S1 lists the countries with the top 10 publications. Among them, China had the highest publications, with a total of 980 articles published, followed by Japan (*N* = 66) and the United States (*N* = 57). Countries with more than 10 publications were Iran, Australia, South Korea and Germany, the remaining countries had either 10 publications or no previous publications. As for the average citation, the highest ranking was in the order of Japan (*N* = 65.20), Australia (*N* = 49.71), South Korea (*N* = 38.18). To gain further insight into regional cooperation in publications, we conducted a co-authorship analysis and visualized the results. In Figure 3A, it can be seen that Asia, represented by China and Japan, and North America, represented by the United States, became the center of the current research, countries from other continents also participated in the study through direct or indirect collaboration. Figure 3B further validates this claim through a cooperation network of the world map.

A total of 793 organizations worldwide had participated in the field. Zhengzhou University had the largest number of publications (*N* = 118), followed by Nanjing Medical University (*N* = 110) and Hebei Medical University (*N* = 55). We included in Supplementary Table S2 the organizations with the top 10 publications, all of them were Chinese institutions. We performed co-authorship analysis in order to better understand publication cooperation between organizations (Figure 3C). Compared with other countries, the institutes within China were numerous and more cooperative. Among them, Zhengzhou University, Nanjing Medical University, and Hebei Medical University were the most active in cooperative research.

### The co-authorship analysis of authors and co-cited authors

**FIGURE 4:**
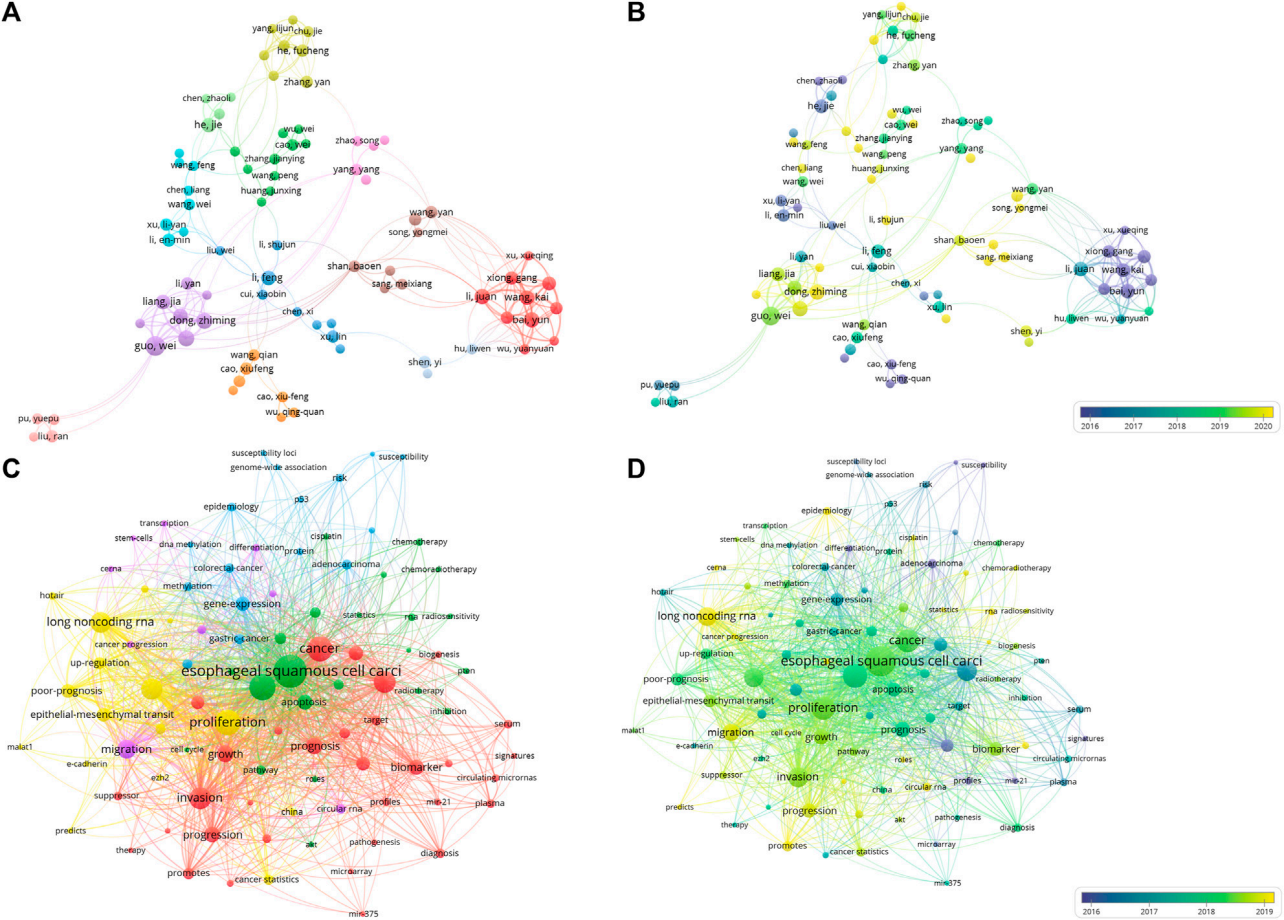
The co-authorship analysis and co-occurrence analysis of ncRNAs associated with ESCC. **(A)** Network plot of co-authorship analysis of authors. **(B)** Overlay map of co-authorship analysis of authors. **(C)** Network plot of co-occurrence analysis of top 100 keywords. **(D)** Overlay map of co-occurrence analysis of top 100 keywords. In network plot, a circle represents an author or a keyword. The larger the circle, the more articles the author has published or the more frequently the keyword appears. The thickness of the connecting line between circles represents the strength of the association. In overlay map, cold colors indicate that the author was active earlier or the keywords appeared earlier, while warm colors indicate that the author was active more recently or the keywords appeared more recently.

A total of 5,426 authors took part in the relevant research over the past 15 years. We listed the top 10 high productive authors in Supplementary Table S3. The first three scholars, all from Hebei Medical University, were Guo Wei, Dong Zhiming, Guo Yanli. It also can be found that these high-yielding scholars were all from China, suggesting the important contributions made by Chinese scholars in the current field. To reveal the contribution and cooperation of authors, the co-authorship analysis of top 100 authors was conducted (Figure 4A). By visualizing the network according to the mean publication year, two largest clusters can be found to be representative: the earlier conducted studies by the red cluster have authors Bai Yun, Li Juan, and Wang Kai, while the currently active purple cluster in the field have authors Guo Wei, Dong Zhiming, and Guo Yanli (Figure 4B).

By analyzing co-cited authors, we learn about the impact and contribution of authors in this academia, as well as examining interdigitation and collaboration in different disciplinary areas. Supplementary Table S3 demonstrates the top 10 co-cited authors. The first three authors were David P Bartel from Howard Hughes Medical Institute (United States ), George A Calin from Ohio State University (United States ), and Chen Wanqing from National Cancer Center (China). It can be found that most of these authors were from the United States, illustrating the founder contribution that American scholars have made to the development of this field. Furthermore, the authors come from different fields, embodying that modern scientific research is the product of the intersection of different disciplines.

### The distribution of source journals and highly cited articles

A total of 261 journals had published articles on ncRNAs associated with ESCC. Among them, *Oncology Letters* published the most article (*N* = 31), followed by *Oncotarget* (*N* = 30), *Oncology Reports* (*N* = 28). From the total citations, the number one was *Oncotarget* (*N* = 1958), followed by *Tumor Biology* (*N* = 719) and *Oncology Reports* (*N* = 623). Supplementary Table S4 lists the top 10 journals with the largest number of publications. Of these, one journal had an impact factor greater than 5: *Biomedicine & Pharmacotherapy* (IF = 7.419). Additionally, *Oncotarget* and *International Journal of Clinical and Experimental Pathology* had no impact factor because they were not currently included in the SCIE database since 2018 and 2020, respectively.

The citations of a paper can reflect its impact and visibility in the field to some extent. The total number of citations for the 1,118 articles were 30,340, with an average of 29 citations per article. The top 10 most cited articles are shown in Supplementary Table S5, with frequencies ranging from 237 to 551. These articles were published between 2008 and 2015, with the 2008 publication titled *Distinctive microRNA profiles relating to patient survival in esophageal squamous cell* carcinoma being the earliest in the field, the prelude to the research on ncRNAs associated with ESCC has since been revealed. Articles titled *Circular RNA ITCH has inhibitory effect on ESCC by suppressing the Wnt/β-catenin pathway*, published in 2015, was the most highly cited with up to 551 citations. Furthermore, these articles were authored by scholars from Japan and China, with 7 articles authored by Chinese scholars and 3 articles authored by Japanese scholars.

### Keywords analysis

We conducted cluster analysis of the top 100 keywords according to the frequency of their occurrence (each keyword appeared at least 12 times). In Figure 4C, analyzing the clusters reveals that the 100 keywords are mainly divided into five clusters. The red cluster mainly includes keywords such as microRNA, biomarker, tumor-growth, prognosis and invasion, this suggests that miRNAs are potential biomarkers for ESCC, and their expression levels correlate with the occurrence and development of ESCC. Using these miRNAs as biomarkers could aid in the diagnosis and treatment of ESCC. The green cluster mainly includes ESCC, cancer cell, apoptosis, autophagy, mechanism, radiosensitivity, uncovering the cellular and molecular mechanisms by which ncRNAs participate in the development and progression of ESCC, and the association between ncRNAs and radiotherapy sensitivity of ESCC. The yellow cluster mainly includes long non-coding RNAs, metastasis and EMT, indicating the important roles and regulatory mechanisms of lncRNAs in the development and progression of ESCC. The purple cluster mainly includes migration, circular RNA and ceRNA, this suggests that circRNAs as ceRNA (competing endogenous RNA) involved in the development and progression of ESCC. The blue cluster mainly includes gene-express, DNA methylation, protein and susceptibility loci, these have implicated related molecular mechanisms in the regulation of ncRNAs in ESCC.

**FIGURE 5:**
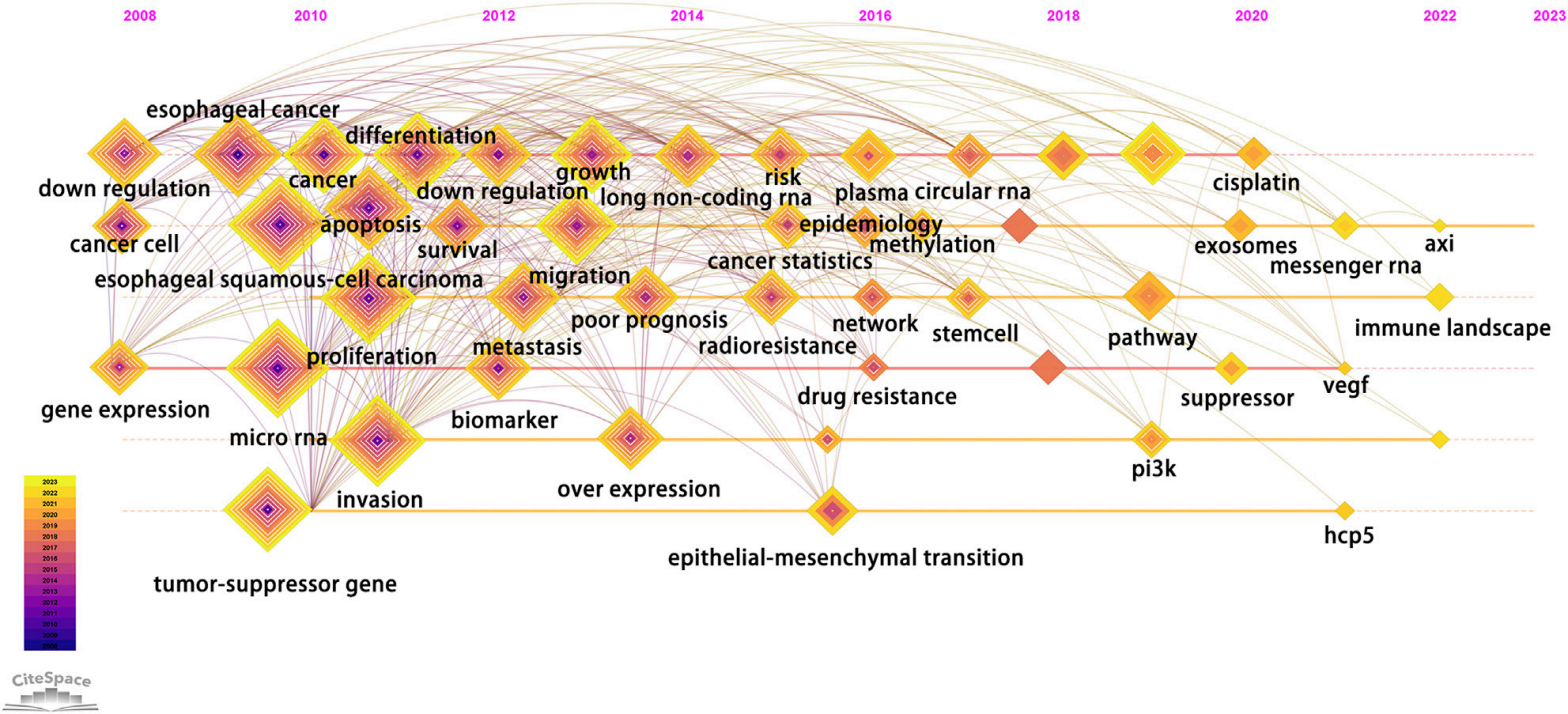
Timeline chart of keywords. Each diamond represents a keyword, and its position on the timeline represents the time it appeared. In addition, the size of the diamond represents the number of times it appears in the total time period, while each layer inside the diamond represents the number of times it appears in the corresponding year.

**FIGURE 6:**
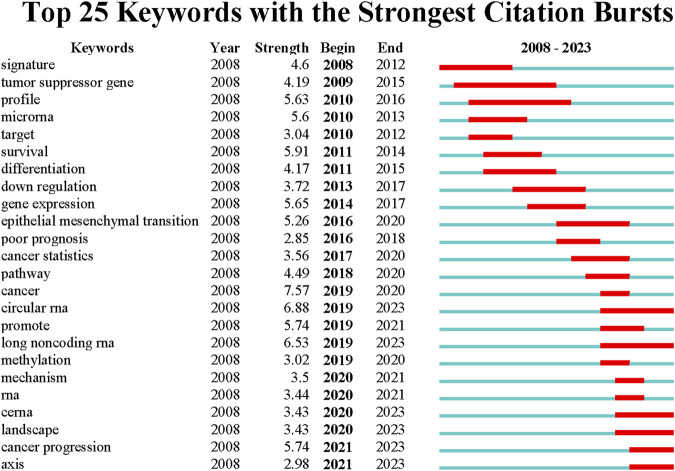
Top 25 keywords with the strongest citation burst. Blue lines indicate the time period in which the keywords appeared and ended, and red lines indicate the time period in which the keywords at high frequencies.

According to the average publication year, overlay visualization of the co-occurrence network of 100 keywords can reveal the prevalence trends of keywords. Cool colors denote earlier published keywords, whereas warm colors indicate later ones. As shown in Figure 4D, it is not difficult to find that the earliest keywords to appear were polymorphism, susceptibility, tumor-suppressor gene, profiles, mir-21, signatures, microarray, and the latest keywords were ceRNA, cancer progression, circular RNA, exosome. To explore the variation and trend of keywords in this field, we plotted a timeline for the high-frequency keywords that appear each year. As shown in Figure 5, It can be found that the early focus of this field was on the expression profile of ncRNAs, as well as the biological process of ncRNAs on the differentiation and apoptosis of ESCC cells, and on predicting the survival of ESCC patients. At this point, the main focus of attention on ncRNAs was miRNAs (2008–2012). In the mid-term stage of this field, more attention had been paid to the mechanisms by which ncRNAs participate in the occurrence and development of ESCC, including EMT, migration, etc. In addition, attention had been paid to the role of ncRNAs in the treatment and prognosis of ESCC. During this stage, lncRNAs gradually gained attention (2012–2016). At the present stage, based on the long-term research on the role of ncRNAs in the occurrence, development, diagnosis, treatment and prognosis of ESCC, circRNAs began to receive attention. And the recent keywords in this field were vegf, pi3k and hcp5 (2016–2022). In order to further understand the outbreak range of hotspots, we identified the top 25 keywords with the strongest citation burst in Figure 6. Focusing on these keywords, it can be observed that researchers have extensively concentrated on the role of ncRNAs in the development, progression, and prognosis of ESCC, along with the related mechanisms of their regulation. It can also be seen that the earliest ncRNAs that had received attention were miRNAs with the citation burst in 2010–2013, while circRNAs and lncRNAs are the current and future research trend with the citation burst in 2020–2023.

## Discussion

Our study delved into the research of ncRNAs associated with ESCC through a bibliometric analysis that reviewed 1,118 relevant articles indexed in the SCIE database from 2008 to 2023. We scrutinized annual publication trends, affiliations, authors, co-cited authors, source journals, highly cited articles, keywords, and further investigated the current development trends and hot topics in this area.

### General overview

By analyzing the trends in annual publications, the development of the research field can be divided into three phases: slow rise in the early stage (2008–2012), rapid rise stage (2013–2019), and stagnation in the later stage (2020–2023) (Figure 2). The trends in the first two stages may be attributed to the development of high-throughput sequencing technology. The article titled *Distinctive microRNA profiles relating to patient survival in esophageal squamous cell carcinoma* published by Guo Y et al. was the first publication in the field and one of the most highly cited articles [13] (Supplementary Table S3). The authors used advanced microRNA microarray technology to study the expression of miRNAs in esophageal cancer tissue and identified 46 RNAs that were specifically expressed in tumor tissues. Thereafter, lncRNAs and circRNAs expression profiles in ESCC were sequentially revealed [14, 15]. Building on previous work from these studies, researchers can move to deeper studies around ncRNAs and ESCC. For example, mining sequencing results through bioinformatics technology and annotating differential genes can help reveal the molecular mechanism of ncRNAs in ESCC [16, 17]. Another possible reason why research on ncRNAs in ESCC had been so popular is that ncRNAs are numerous of the transcriptome and are involved in many cellular processes [3], yet previous research in this area has been lacking. Therefore, it attracted the attention from researchers. However, the third stage of annual publications perhaps indicating that research in the field had encountered bottlenecks. The possible explanations for why there is a trend towards stagnation when it comes to investigating the mechanism of ncRNAs in ESCC could be several. Firstly, previous studies had exhaustively investigated the role of ncRNAs in ESCC, leading to the exhaustion of the field. Secondly, the research conducted so far has almost been limited to basic research, which had hindered the translation of the research results into clinical applications. Thirdly, the ncRNAs mechanism in ESCC is complex, and the field is striving to find new insights into this diverse mechanism. However, the current research model is tending towards homogenization.

In this field, China had the largest number of organizations and the most prolific authors engaged in relevant research, which had resulted in the highest productions. Japanese papers had the highest average citation rate, while the greatest number of co-cited authors cited were from the United States (Supplementary Tables S1–S3). This indicated that China was at the forefront in terms of the high yield in the field. Japanese and American research achievements in this field had a high level of influence and recognition. It can be explained by the fact that Japan and China are high-risk countries for esophageal cancer, both countries attach great importance to research on esophageal cancer [1]. And the United States holds strong strength for biomedical research [18]. In addition, the Matthew effect can also be used as a possible explanation, as these high-yielding countries have more output compared to other countries with fewer resources due to their existing research funding support or research capabilities [19]. It is worth noting that despite having the highest publication volume, China’s average citation of papers was not optimistic, indicating the need for further efforts to enhance the quality of research. As shown in Figure 3, 25 countries had participated in this research area, and a research center has been formed with China, Japan, and the United States, but the situation is that there was more frequent domestic cooperation in each respective country. The reason for this phenomenon may be that language and cultural differences between different countries may cause difficulties in communication and cooperation. On the other hand, research institutions and scientists in different countries may have varying resource and interest needs, leading to difficulties in balancing cooperation between countries. Therefore, we also look forward to closer cooperation between countries in order to produce more high-quality research.

Based on publications and citations, *Oncotarget* was the most influential journal in this field (Supplementary Table S4). However, the journal was not indexed by SCIE in 2018 due to excessive self-citation and potentially unjustified co-authored research. Similarly, *International Journal of Clinical and Experimental Pathology* was not indexed by SCIE in 2020. After excluding these two journals, *Tumor Biology* had the highest average citation count, establishing it as the most influential journal in this field. It is an open access journal focused on tumor related research, which also suggests that open access journals have become increasingly popular in recent years as they provide free and unrestricted access to research articles, making it easier for researchers and academics to access and share knowledge. This has helped to increase the visibility and impact of research and has also promoted greater collaboration and exchange of ideas within the academic community. Therefore, many researchers and institutions are now recognizing the value of open access publishing and are choosing to publish their research in open access journals. Highly cited articles, to some extent, also reflect hot topics of concern in the field of scientific research, and their published journals also have an undeniable influence in this field. In addition to the aforementioned journals, the articles published in the following journals rank among the top ten in terms of citation: *International Journal of Cancer, Cancer, Gut, Cancer Research, Molecular Cancer, Clinical Chemistry, Journal of Biological Chemistry, and Clinical Cancer Research.* These journals are positioned in fields such as oncology, molecular biology, pharmacy, and clinical medicine, reflecting the long-standing and future scientific research model of multidisciplinary cooperation.

### Research hotspots

We employed co-occurrence analysis of keyword networks (Figures 4C, D) and timeline charts (Figure 5) to unveil the distribution of research focus in the field. Additionally, we analyzed keywords with the strongest citation burst to further uncover the evolving trends of research hotspots (Figure 6). Based on the above results, we can divide the research process in this field into three parts: 1. ncRNAs as a diagnostic and prognostic marker of ESCC; 2. ncRNAs participates in the occurrence and development of ESCC; 3. the therapeutic potential of ncRNAs on ESCC. The main studied ncRNAs include miRNAs, lncRNAs, and circRNAs.

One of the most promising research parts for ncRNAs in ESCC is its potential as a diagnostic and prognostic biomarker, which has been highly valued since the beginning of research in this field. Compared to normal physiological conditions, the levels of specific ncRNAs produced by cancer cells may undergo significant alterations. The detection of their expression can yield valuable biological information [20]. Furthermore, certain ncRNAs exhibit widespread stability in bodily fluids, such as blood, saliva, and urine [21]. This forms the basis for efficient, non-invasive, and cost-effective detection methods Studies have demonstrated the utility of ncRNAs as prognostic and diagnostic biomarkers for ESCC, with miRNAs being the most well-studied in this regard. For example, studies have shown that high expression of miR-1246, miR-766-3p, miR-20b-5p, and miR-1290 in the serum of ESCC patients has strong diagnostic and prognostic potential [22–25]. Similarly, lncRNAs such as HOTAIR, MALAT1, CCAT2, and PCAT-1, and circRNAs such as circ-SLC7A, circ-SMAD7, circ_000194, and circ-GSK3β, have also been identified as potential diagnostic and prognostic biomarkers for ESCC [15, 26–32]. In addition, there is a study that has taken a step further towards the identification of miRNAs (miRNA-103, miRNA-106b, miRNA-151, miRNA-17, miRNA-181a, miRNA-21, miRNA-2 and miRNA-93) with diagnostic implications in a retrospective and prospective multicenter cohort study. The results demonstrate higher specificity and sensitivity of miRNAs markers compared with traditional tumor markers including SCC Ag, CEA and CYFRA21-1, further confirming the practical significance of serum miRNAs used as diagnostic markers for early-stage ESCC [33].

Another part of research on ncRNAs in ESCC is their role in the initiation and progression of the disease. Studies have shown that ncRNAs can act as tumor promoters or suppressors in ESCC by regulating several biological processes such as proliferation, metastasis, apoptosis, cell cycle, DNA damage repair, and EMT [34–37]. Many miRNAs play a role by binding to the 3′UTR of the target gene, blocking its translation and leading to its inactivation at the mRNA level, or participating in the related pathways of G protein coupled receptors [38–40]. LncRNAs can recruit chromatin remodeling factors or inhibit transcription factors of target gene promoters to exert its effects [41]. Recently, it was found that lncRNAs can regulate the expression of related miRNAs and then participate in the occurrence and development of ESCC. For example, lncRNA FAM83A-AS1 downregulated the expression of miRNA-214, which indirectly deregulated the repression of CDC25B by miRNA-214, and thus participated in ESCC progression [42]. Similarly, circRNAs such as circRUNX1, ciRS-7, and LPAR3 function as ceRNAs of their counterpart miRNAs, contributing to the development of ESCC [4, 43, 44]. The term “ceRNA” refers to the ability of circRNAs to serve as miRNA sponges by sequestering them and inhibiting their regulatory effects on downstream targets, thus exerting their own regulatory roles.

The third part of research on ncRNAs in ESCC is their potential for use in treatment. NcRNAs may be able to improve the efficacy of existing therapies, such as chemotherapy and radiotherapy, or act as novel therapeutic targets. The main chemotherapy regimen for ESCC patients mainly includes cis-diamminedichloro-platinum (CDDP), 5-fluorouracil, and gefitinib. If the patient is not sensitive to these anticancer drugs, it may lead to unsatisfactory treatment effectiveness [45–47]. Studies have shown that high-risk ncRNAs, such as miR-624, miR-140-3P, lncRNA NORAD, lncRNA CASC8, and hsa_circ_0000277, can increase chemotherapy resistance in ESCC patients. Therefore, reversing the expression of high-risk ncRNAs may have a positive effect on reducing chemotherapy resistance, thereby improving treatment efficacy [5, 6, 48–50]. For instance, re-expression of circRNA-cdopey2 decreased the amount of anti-apoptotic protein Mcl-1 expression, which in turn enhanced the apoptosis of ESCC cells and enhanced the killing ability of CDDP on cancer cells [51]. Jin et al. showed that the overexpression of miR-141-3p can promote acquired chemotherapy resistance of 5-fluorouracil by regulating tumor suppressor PTEN [46]. In addition, lncRNAs (such as AFAP1-AS1 and LINC01014) participate in Wnt/β-Catenin and PI3K/Akt/mTOR signaling pathways reduced the effectiveness of chemotherapy drugs including gefitinib [52]. Radiotherapy is a radical treatment modality for ESCC, but studies have found that ncRNAs are involved in the sensitivity of ESCC cells to radiation. Dysregulation of ncRNAs such as miRNA-4443 and miRNA-21 can affect cell cycle, apoptosis, and DNA damage repair, making them potential targets for improving ESCC radiosensitivity [37, 53]. The abundance of miRNA-21 can be decreased by upregulating lncRNA gas5, which further upregulates expression of a transformation repressor named RECK to enhance ESCC cell radiosensitivity [53]. Similarly, circvrk1 was found to be under expressed in ESCC tissues and cell lines compared with controls. Radiation resistance of ESCC cells could be alleviated by overexpression of circvrk1, the mechanism is that circvrk1, as the ceRNA of mir-624-3p, upregulates PTEN and exhibits a role in reducing radiation resistance [54]. In summary, ncRNAs have great potential in the field of ESCC treatment. Unfortunately, as of now, there are no large-scale, high-quality randomized controlled clinical studies to further validate.

With the emergence of new technologies, research on ncRNAs in ESCC has been further explored based on previous research models. For example, relevant research focuses on ncRNAs in exosomes, because as a subgroup of tumor microenvironment, it promotes cell communication by transmitting various biological molecules [55]. Exosomes carrying ncRNAs, due to their stability and widespread presence in body fluids, along with advancements in exosome separation technology, are expected to make the detection of ncRNAs for screening ESCC patients non-invasive, convenient, and efficient [56]. In addition, studies have also focused on the immune landscape related to ncRNAs in ESCC, which will be of great significance in revealing the tumor immune microenvironment. The continuous evolution of research on the immune landscape has given rise to a variety of novel technologies, providing more in-depth and comprehensive insights. These encompass single-cell RNA sequencing, bioinformatics, and machine learning. The integrated application of these emerging technologies enables researchers to gain a more comprehensive and profound understanding of the intricacies of the immune landscape, particularly in relation to ncRNAs [57, 58].

Therefore, it can be said that the study of ncRNAs in ESCC has opened up new avenues for the development of novel diagnostic, prognostic, and therapeutic approaches. Our study can also provide insights into the focus of future studies. On the one hand, future studies can further apply new technologies (e.g., Mendelian randomization and network pharmacology) to explore the role of ncRNAs in ESCC based on current research model. On the other hand, more in-depth studies, especially clinical studies are needed to validate the value of ncRNAs in the practical clinical application of ESCC.

### Limitation

Our study utilized bibliometrics to comprehensively analyze the progression of research on ncRNAs associated with ESCC. However, we must be aware of potential limitations. Firstly, considering the completeness and authenticity of the data obtained, we used only the SCIE database to retrieve related articles. Secondly, quality assessment was not conducted due to the large volume of included literature, potentially leading to variations in article quality. Thirdly, due to the unavoidable issue of inconsistent spelling or identical names among authors, the author names automatically extracted by software may differ slightly from the actual situation, and currently, there is no effective method for bulk correction.

### Conclusion

In summary, exploring the roles and mechanisms of ncRNAs in ESCC has paved the way for the discovery of novel diagnostic, prognostic, and therapeutic approaches. However, the current research paradigm in this field tends towards homogeneity. Future investigations should focus on providing more nuanced explanations for the functions of ncRNAs, aiming to gain a deeper understanding of its role in ESCC. This involves a concerted effort to translate these findings into clinical research, validating the specificity, sensitivity, and clinical feasibility of ncRNAs in ESCC. Integrating novel interdisciplinary elements into the existing research framework or shifting the research emphasis from preclinical studies to clinical investigations centered on diagnosis, treatment, and prognosis will likely constitute the primary avenues for breakthroughs in this field.

## Data Availability

The data used in this study comes from published studies, and all data related to the research results are included in the main text. For more information, please consult the corresponding authors.
