# Supplementary material for: Comprehensive landscape and future perspectives of non-coding RNAs in esophageal squamous cell carcinoma, a bibliometric analysis from 2008 to 2023
Source: Pathol Oncol Res. 2024 Feb 21;30:1611595. doi: 10.3389/pore.2024.1611595 (PMC10915033; doi:10.3389/pore.2024.1611595)
Supplement: Supplementary file 1 [file DataSheet1.DOCX]

| **Rank** | **Country/region** | **Documents** | **Citations** | **Average citation/publication** |
| --- | --- | --- | --- | --- |
| 1 | China | 980 | 24677 | 25.18 |
| 2 | Japan | 66 | 4303 | 65.20 |
| 3 | The United States | 57 | 1395 | 24.47 |
| 4 | Iran | 15 | 314 | 20.93 |
| 5 | Australia | 14 | 696 | 49.71 |
| 6 | South Korea | 11 | 420 | 38.18 |
| 7 | Germany | 11 | 359 | 32.64 |
| 8 | Taiwan, China | 8 | 238 | 29.75 |
| 9 | India | 7 | 106 | 15.14 |
| 10 | England | 6 | 85 | 14.17 |

**Table S1.** Top 10 productive countries/regions

| **Rank** | **Organization** | **Country** | **Documents** | **Citations** | **Average citation/publication** |
| --- | --- | --- | --- | --- | --- |
| 1 | Zhengzhou University | China | 118 | 2892 | 24.51 |
| 2 | Nanjing Medical University | China | 110 | 4355 | 39.59 |
| 3 | Hebei Medical University | China | 55 | 932 | 16.95 |
| 4 | Sun Yat Sen University | China | 42 | 1933 | 46.02 |
| 5 | Chinese Academy of Medical Sciences | China | 40 | 2291 | 57.28 |
| 6 | Soochow University | China | 38 | 1515 | 39.87 |
| 7 | Shandong University | China | 29 | 770 | 26.55 |
| 8 | Peking Union Medical College | China | 26 | 1848 | 71.08 |
| 9 | Capital Medical University | China | 25 | 478 | 19.12 |
| 10 | Nanjing University | China | 24 | 852 | 35.50 |

**Table S2.** Top 10 productive organizations

| **Rank** | **Author** | **Institution(country)** | **Documents** | **Citations** | **ACI** | **Co-cited author** | **Institution(country)** | **Co-citations** |
| --- | --- | --- | --- | --- | --- | --- | --- | --- |
| 1 | Guo Wei | Hebei Medical University  (China) | 25 | 479 | 19.16 | David P Bartel | Howard Hughes Medical Institute  (USA) | 191 |
| 2 | Dong Zhiming | Hebei Medical University  (China) | 18 | 272 | 15.11 | George A Calin | Ohio State University (USA) | 126 |
| 3 | Guo Yanli | Hebei Medical University  (China) | 17 | 292 | 17.18 | Wanqing Chen | National Cancer Center  (China) | 156 |
| 4 | Bai Yun | The Third Military Medical University  (China) | 16 | 1077 | 67.31 | Peter C Enzinger | Dana-Farber Cancer Institute  (USA) | 139 |
| 5 | Li Juan | The Third Military Medical University  (China) | 16 | 869 | 54.31 | Jacques Ferlay | International Agency for Research on Cancer  (France) | 100 |
| 6 | Wang Kai | The Third Military Medical University  (China) | 15 | 1026 | 24.00 | Ahmedin Jemal | American Cancer Society  (USA) | 225 |
| 7 | Liang Jia | Hebei Medical University  (China) | 15 | 286 | 19.07 | Yang Li | National University of Singapore  (Singapore) | 105 |
| 8 | He Jie | Chinese Academy of Medical Sciences  (China) | 14 | 836 | 59.71 | K J Livak | Applied Biosystems  (USA) | 118 |
| 9 | Shen Supeng | Hebei Medical University  (China) | 14 | 276 | 19.71 | Arjun Pennathur | University of Pittsburgh School of Medicine  (USA) | 146 |
| 10 | Li Feng | Capital Medical University  (China) | 14 | 208 | 14.86 | Rebecca L Siegel | American Cancer Society  (USA) | 104 |

**Table S3.** Top 10 productive authors and co-cited authors

**ACI**: average citation per item

| **Rank** | **Journal** | **IF** | **JCR** | **Documents** | **Citations** | **ACI** |
| --- | --- | --- | --- | --- | --- | --- |
| 1 | Oncology Letters | 3.111 | Q3 | 31 | 414 | 13.35 |
| 2 | Oncotarget | NA | NA | 30 | 1958 | 65.27 |
| 3 | Oncology Reports | 4.136 | Q3 | 28 | 623 | 22.25 |
| 4 | International Journal of Clinical and Experimental Pathology | NA | NA | 25 | 572 | 22.88 |
| 5 | European Review for Medical and Pharmacological Sciences | 3.784 | Q2 | 25 | 458 | 18.32 |
| 6 | Oncotargets and Therapy | 4.345 | Q2 | 23 | 480 | 20.87 |
| 7 | Molecular Medicine Reports | 3.423 | Q3 | 23 | 264 | 11.48 |
| 8 | Tumor Biology | 3.650 | Q2 | 17 | 719 | 42.29 |
| 9 | Biomedicine & Pharmacotherapy | 7.419 | Q1 | 17 | 448 | 26.35 |
| 10 | Cancer Management and Research | 3.602 | Q3 | 17 | 207 | 12.18 |

**Table S4.** Top 10 productive journals

**IF**: impact factor, **JCR**: journal citation reports, **ACI**: average citation per item

| **Rank** | **Title** | **Authors** | **Journal** | **Year** | **Citations** |
| --- | --- | --- | --- | --- | --- |
| 1 | Circular RNA ITCH has inhibitory effect on ESCC by suppressing the Wnt/β-catenin pathway | Li F, Zhang L, Li W, et al. | Oncotarget | 2015 | 551 |
| 2 | miR-145, miR-133a and miR-133b: Tumor-suppressive miRNAs target FSCN1 in esophageal squamous cell carcinoma | Kano M, Seki N, Kikkawa N, et al. | International Journal of Cancer | 2010 | 408 |
| 3 | Clinical impact of serum exosomal microRNA-21 as a clinical biomarker in human esophageal squamous cell carcinoma | Tanaka Y, Kamohara H, Kinoshita K, et al. | Cancer | 2013 | 342 |
| 4 | LncRNA profile study reveals a three-lncRNA signature associated with the survival of patients with oesophageal squamous cell carcinoma | Li J, Chen Z, Tian L, et al. | Gut | 2014 | 308 |
| 5 | Distinctive microRNA profiles relating to patient survival in esophageal squamous cell carcinoma | Guo Y, Chen Z, Zhang L, et al. | Cancer Research | 2008 | 305 |
| 6 | Identification of the long non-coding RNA POU3F3 in plasma as a novel biomarker for diagnosis of esophageal squamous cell carcinoma | Tong YS, Wang XW, Zhou XL, et al. | Molecular Cancer | 2015 | 278 |
| 7 | Expression profile of microRNAs in serum: a fingerprint for esophageal squamous cell carcinoma | Zhang C, Wang C, Chen X, et al. | Clinical Chemistry | 2010 | 263 |
| 8 | Silencing of long noncoding RNA MALAT1 by miR-101 and miR-217 inhibits proliferation, migration, and invasion of esophageal squamous cell carcinoma cells. | Wang X, Li M, Wang Z, et al. | Journal of Biological Chemistry | 2015 | 253 |
| 9 | MicroRNA-10b promotes migration and invasion through KLF4 in human esophageal cancer cell lines | Tian Y, Luo A, Cai Y, et al. | Journal of Biological Chemistry | 2010 | 253 |
| 10 | MicroRNA-21 regulates the proliferation and invasion in esophageal squamous cell carcinoma | Hiyoshi Y, Kamohara H, Karashima R, et al. | Clinical Cancer Research | 2009 | 237 |

**Table S5.** Top 10 high cited articles
